# The Diagnosis of Scarlet Fever
1A Paper read at the meeting of the Bath and Bristol Branch of the British Medical Association at Weston-super-Mare, May, 1925.


**Published:** 1925

**Authors:** John Wallace

**Affiliations:** Medical Officer of Health and Medical Superintendent, Isolation Hospital, Weston-super-Mare


*" Scire est nescire, nisi id me*
*Scire alius scierit."*

AUTUMN, 1925.

### THE DIAGNOSIS OF SCARLET FEVER.[1]

BY

JOHN WALLACE, O.B.E., M.B., C.M. Ed.,

*Medical Officer of Health and Medical Superintendent, Isolation Hospital, Weston-super-Mare.*

In the beginning of last century Heberden the elder wrote of scarlet fever: " This distemper is sometimes so slight as to require no remedies, and sometimes so violent as to admit of no relief." As it was then so it is to-day, but fortunately the general run of cases is of the less serious type and the mortality is comparatively low.

The history of the disease, however, shows that its severity has lessened and again become aggravated in cycles of varying length, and it may be that we are only passing through a period of milder attacks which is only temporary. Our cases in Weston-super-Mare are too few to base any generalisations on them, but after some eight or ten years

[1] A Paper read at the meeting of the Bath and Bristol Branch of the British Medical Association at Weston-super-Mare, May, 1925.

of a mild type of the disease we have had during the past year several cases of a toxic type and a much greater tendency to "complications" occurring in others; this may be the beginning of a new cycle.

That scarlet fever may be still of great severity when it attacks virgin soil is seen in a report by Monillac in connection with an outbreak lasting from March, 1921, to May, 1922, in Yunnan in South China, where the disease had not existed in living memory. About 50,000 persons, or nearly one quarter of the population, appear to have died; eighteen Europeans were attacked, and all recovered.

It is obvious that a disease of this character is of serious importance from the point of view of public health. It has long been recognised as a dangerous infectious fever, and in this country is "notifiable," and in many sanitary districts the local authorities provide hospital accommodation for cases of scarlet fever and other diseases with which they may be confused. Correct diagnosis of the disease is therefore of special importance if cross-infection is to be avoided.

Squire defines scarlet fever as "an infectious specific fever, with sudden ingress, soreness and redness of the throat, and a finely-diffused scarlet rash on the second day, most intense on the third day, beginning to fade on the fifth or sixth with some subsidence of the fever, and followed by desquamation of the cuticle, in both small and large flakes; and inducing a liability to rheumatic and renal symptoms and a tendency to serious effusions." That is a sufficiently comprehensive description of what one has to look for and would probably find in a typical case of average severity, and the diagnosis would be plain sailing.

In ordinary measles (with which till about the middle of the sixteenth century scarlet fever was confused) typical cases seem to be the rule and the diagnosis easy, but scarlet fever "is so varied in its manifestations and liable on occasion to assume such mild forms that the diagnosis is often extremely difficult." Even in the severe form in the Yunnan epidemic it took on a marked anginose type, and was at first mistaken for and treated as diphtheria. The rash also was frequently absent.

Our chief difficulty in Weston is with the very mild types of scarlet fever, where the local signs are slight and constitutional symptoms practically absent. I am frequently asked to see such cases in consultation, and often the household conditions make a decision on the case one of very considerable responsibility, which the practitioner in attendance is very wise to share with someone else. In particular one has only to mention a suspicious case arising in one of our many boarding schools to get you to visualise how many different interests may be affected by the diagnosis, while if a definite diagnosis is not possible the worries and inconveniences of quarantine are far from negligible.

How often one has wished for some infallible simple test. There was the *Rumpel-Leede* sign—a bandage round the upper arm stopping the venous return but not the arterial pulse was said to produce after a few minutes petechial hemorrhages in the fold of the elbow in a true case of scarlet fever. Well, it did not always come off when it was most wanted, and it also occurred in other exanthemata, so I gave it up.

So also with the *Diazo reaction* in the urine and "Dohle's" inclusion bodies in polymorph leukocytes.

I have not tried the "Schultz-Charlton" or extinction sign, but even if it were definite I think you will agree that a Wassermann reaction and a test of the serum for sterility would be essential before injecting a patient, and would prevent any wide use of the test.

Naturally we have all been looking to the bacteriologist to help us as he has done in diphtheria, enterica, etc., by discovering an easily recognisable and readily procurable germ as the guilty agent of the disease. Klein, Mervyn Gordon and others have worked at the streptococci found in the throats and secretions of scarlet fever patients for many years, and in particular have isolated the *S. hæmolytica*, but have failed to prove its specific relation to the chief manifestations of scarlet fever. Recently, however, the Dicks of Chicago appear to have had more success. Dr. Copeman, of the Ministry of Health, paid an official visit to New York to investigate some of their work, which he described on April 23rd last at a meeting of the Royal Society of Medicine. He has been so impressed with the work that the Ministry has appointed a Medical Officers' Committee on scarlet fever, and that Committee recommends further study of the questions raised by the Dicks' conclusions. But I gather that there is real difficulty in differentiating the offending member of the hæmolytic streptococci, and that the streptococcus has not always been found in the throat of scarlet fever cases.

A few years ago I took a child into hospital for scarlet fever, and found she had faucial diphtheria as well. She probably had been an old-standing carrier, for she continued to have positive swabs long after she had ceased desquamating and had quite recovered from the attack of scarlet fever. At the end of nine weeks the ward she occupied alone was required for other patients, so she was carefully examined and found to be free from any apparent sequelæ. She had the usual discharge bath and, wearing clothing previously stoved, was placed in the only available ward with a little boy recovering from diphtheria. On the third day the boy developed typical scarlet fever. Dr. Savage kindly saw the girl with me and took swabs from the nose and throat on the possibility of finding streptococci present, but the result of the bacteriological examination was negative.

How, then, is one to come to a decision in the abnormally mild cases so long as the bacteriologist is baffled in his efforts to help us ?

Heubner of Berlin lays great stress on the rash—its peculiar characteristic finely, delicately punctuate appearance, rose-red at first but gradually darkening and coalescing till it assumes a flaming, fire-red look justifying the name of scarlet fever. The punctiform character may not be universal, but it can generally be seen on some area of the body. But he admits the rash may be absent, and then the diagnosis may become very difficult.

Dr. Duke, on the other hand, advised one not to look at the rash at all until all the symptoms and other signs have been studied along with the history of the case. The sight of the rash is apt to prejudice you, and should only be used as a confirmation of the diagnosis you have arrived at. I agree with this method rather than Heubner's, and if constantly followed it must be a great aid in diagnosing the cases where no rash is to be found at the time of the examination and give confidence to one in his work.

One high authority on the diagnosis of small-pox teaches that when a patient comes under observation you should "avoid letting him or anyone speak to you about the history of the case until you have completed your own examination" (Wanklyn). "To be groping about for a history is to lean upon rotten supports which you are better without." At the same time, he would not shut his ears to a story which makes a *prima facie* case, but would use it as a cause for investigation. I doubt if one should go as far as Wanklyn does as to excluding the history at first in cases of suspected scarlet fever; still, there is much to be said for Sir George Murray Humphry's advice in examining cases in general: "Eyes first, hands next, tongue last and least."

The aspect of the patient is of importance in ordinary cases, for he generally looks, as he is, fairly ill in the early stages ; in the milder cases he may not show any appearance of illness. Then examine the condition of the mouth and pharynx. The *white strawberry* tongue of the second day, the *red strawberry* or *raspberry* tongue of the fourth day, or the *raw beef* tongue which follows are almost invariable phenomena, and the last may persist for a week or more.

In early cases one can generally observe some affection of the throat. Even if you haven't the usual sharply-defined, deep redness there may be slight congestion and some swelling of the tonsils with even a thin veiling deposit on them. The lymph glands at the angle of the lower jaw are generally enlarged and tender on pressure, in many cases painful. The cervical glands may also be enlarged.

Temperature and pulse are noted, and this peculiarity is found in scarlet fever that the rapidity of the pulse in proportion to the height of the temperature and age of the patient is greater than in other diseases :—

*e.g.* 103° F. with 150 to 170 pulse per minute,
102° F. with 130 to 170 pulse per minute,
100° F. with 124 to 170 pulse per minute, even in mild cases in children of five or six years. The trouble of late years has been that temperatures have often been almost normal, but even in these cases an increased pulse-rate is generally found. Albuminuria should be tested for.

If the strawberry tongue and the peculiar pulse-temperature ratio are observed the physician will probably have reached a provisional decision which will be strengthened if one or more of the other signs mentioned are present, and still further supported if he can now get a history of more or less sudden vomiting, and feverishness with headache and general malaise. Often one can get an account of known exposure to possible infection within the maximum incubation period of five days.

Now one can look for a rash; if present and of the character already described there can be no possible doubt of the diagnosis, but its absence need hardly worry one. On the other hand, if it departs from the normal it will require careful study, and if it cannot be brought into line the original diagnosis will have to be revised.

If the result of the first part of one's examination does not tally with the symptoms of scarlet fever then it will seldom be found that the rash is the one which one expects to find in that disease.

If there are no helpful signs but only a suspicious history then the best one can do is to play for safety and treat the case as one of the more dangerous nature and establish temporary quarantine at any rate. A few days may clear things up, or more likely they won't, and one waits hopefully for desquamation—often a broken reed, but all one has to lean on.

If in the course of three weeks or so desquamation is present on the hands and feet, occurs between the fingers and toes and shows a pin-hole type, we can at least feel that the patient's friends may be satisfied, even if we are not, for so many conditions are followed by peeling!

I have said nothing about double infections or two intercurrent diseases, but diphtheria is not seldom found along with scarlet fever, and it is wise to take a swab of the throat before treatment is begun in case Löffler's bacillus be present. Unfortunately, there will always be a number of undecided cases until the *causa causans* is discovered and can be readily diagnosed in the laboratory.

